# Efficacy and Safety of Glucagon‐Like Peptide 1 Receptor Agonists in Parkinson Disease: A Systematic Review and Meta‐Analysis

**DOI:** 10.1002/brb3.71344

**Published:** 2026-03-25

**Authors:** Hassan Raza, Bakhtmeena Nizam, Arif Padaniya, Muhammad Aqib Farid, Amber Nabi, Muhammad Qasim Hasan, Muhammad Salih, Apurva Hiteshkumar Patel, Noora Inam, Mohammad Ebad Ur Rehman, Usman Khan, Asma'a Munasar Ali Alsubari

**Affiliations:** ^1^ Department of Medicine Lahore Medical and Dental College Lahore Pakistan; ^2^ Department of Medicine Women Medical College Abbotabad Pakistan; ^3^ Department of Gastroenterology and Hepatology Cleveland Clinic Cleveland Ohio USA; ^4^ Department of Geriatrics Oxleas NHS Trust London UK; ^5^ Department of Medicine Ziauddin University Karachi Pakistan; ^6^ Department of Medicine Allama Iqbal Medical College Lahore Pakistan; ^7^ Department of Medicine Dow University Hospital Karachi Pakistan; ^8^ Department of Neurology Medical University of South Carolina Charleston South Carolina USA; ^9^ Department of Medicine Gajju Khan Medical College Swabi Pakistan; ^10^ Department of Medicine Rawalpindi Medical University Rawalpindi Pakistan; ^11^ Department of Neurology Geisinger Hospital Scranton Pennsylvania USA; ^12^ Department of Medicine Sana'a University Sana'a Yemen

## Abstract

**Background:**

Parkinson's disease (PD) is a progressive neurodegenerative disorder with no currently approved disease‐modifying treatments. Glucagon‐like peptide‐1 receptor agonists (GLP‐1RAs), originally used in type 2 diabetes, have demonstrated neuroprotective and anti‐inflammatory effects in preclinical PD models. This systematic review and meta‐analysis evaluated the efficacy and safety of GLP‐1RAs in patients with PD.

**Methods:**

A systematic search of MEDLINE, Embase, Cochrane CENTRAL, and ClinicalTrials.gov was conducted through July 2025 for randomized controlled trials (RCTs) comparing GLP‐1RAs to placebo in PD. Primary outcomes included MDS‐UPDRS Part III (motor examination) both on and off medication. Secondary outcomes included MDS‐UPDRS Parts I, II, IV, PDQ‐39, NMSS, and adverse effects. Data were pooled using a random‐effects model with results reported as mean differences (MD) or risk ratios (RR) with 95% confidence intervals (CI).

**Results:**

Five RCTs involving 708 participants were included. No statistically significant differences were found in MDS‐UPDRS Part III scores off medication (MD: –2.00, 95% CI: –4.12 to 0.11, p = 0.06) or on medication (MD: –1.40, 95% CI: –3.42 to 0.62, p = 0.17). Secondary outcomes also showed no significant benefits with GLP‐1RA use. However, GLP‐1RAs were associated with increased gastrointestinal side effects, including nausea (RR: 2.09), vomiting (RR: 4.53), constipation (RR: 1.60), and weight loss (RR: 1.83).

**Conclusion:**

Current evidence does not demonstrate a statistically significant overall benefit of GLP‐1RAs on efficacy outcomes in PD, while gastrointestinal adverse events are increased. More trials are needed to clarify their disease‐modifying potential.

## Graphical abstract

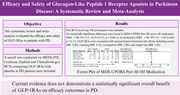

## 1 Introduction

Parkinson's disease (PD) is a progressive neurodegenerative disorder characterized by cardinal motor symptoms—bradykinesia, rigidity, and tremor—as well as a wide range of non‐motor manifestations, including mood disorders, cognitive decline, and gastrointestinal disturbances (Poewe et al. 2017; Bloem et al. 2021). With over 6 million individuals affected worldwide and a projected doubling in prevalence by 2040, PD poses a substantial and growing public health challenge (Dorsey et al. 2018; Kalia and Lang 2015). Despite the availability of symptomatic dopaminergic therapies, current treatment approaches do not modify the underlying disease course and are frequently complicated by motor fluctuations and long‐term complications such as dyskinesias (Armstrong and Okun 2020). This therapeutic gap has fueled interest in alternative pathways and targets with disease‐modifying potential.

Glucagon‐like peptide‐1 receptor agonists (GLP‐1RAs), initially developed for Type 2 diabetes, have demonstrated promising central nervous system effects, including anti‐inflammatory, antioxidant, and neuroprotective actions (Zheng et al. 2024; Santiago and Potashkin 2013; Hölscher 2014). These effects are thought to arise from modulation of shared metabolic and neurodegenerative pathways, such as insulin signaling and mitochondrial function (Elbassuoni and Ahmed 2019; Lv et al. 2024). In animal models of PD, GLP‐1RAs have been shown to reduce dopaminergic neuronal loss, improve behavioral outcomes, and modulate neuroinflammatory markers (Lv et al. 2024).

Building on this mechanistic rationale, several clinical trials have investigated GLP‐1RAs in patients with PD. Early open‐label and double‐blind studies of exenatide demonstrated sustained improvements in motor scores (Mcgarry et al. 2024), while the recent Exenatide‐PD3 and lixisenatide trials extended these findings in larger, placebo‐controlled cohorts (Athauda et al. 2017; Meissner et al. 2024). However, other trials, such as the NLY01 study, failed to meet primary endpoints, highlighting inconsistencies in clinical efficacy (Aviles‐Olmos et al. 2013). A prior meta‐analysis by Messak et al. (2025) reported favorable trends in motor outcomes but was limited by small sample sizes and the absence of more recent large‐scale trials. Furthermore, recent reviews have highlighted potential cognitive benefits of GLP‐1RAs in PD, although these observations await confirmation in dedicated cognitive trials (Lv et al. 2024).

Given the growing yet mixed clinical evidence and the publication of new high‐quality trials in the past 2 years, an updated systematic review and meta‐analysis is warranted. The present systematic review and meta‐analysis aims to synthesize data from recent randomized controlled trials (RCTs), with a focus on both motor and non‐motor outcomes, including safety and tolerability, to clarify the therapeutic potential of GLP‐1RAs in PD.

## 2 Methods

This meta‐analysis was performed according to the guidelines outlined in the Cochrane Handbook for Systematic Reviews of Interventions and reported in accordance with the Preferred Reporting Items for Systematic Reviews and Meta‐Analysis (PRISMA) statement (Cochrane 2024; Page et al. 2021). This review was registered with PROSPERO (CRD420261327531).

### 2.1 Data Sources and Searches

The following electronic databases were used for the literature search: Cochrane Central Register of Controlled Trials, MEDLINE, Embase, and ClinicalTrials.gov from inception till July 2025. Reference lists were also screened to include potentially relevant articles. “Parkinson Disease” and “Glucagon‐Like Peptide‐1 Receptor Agonists” were used as Medical Subject Heading (MeSH) terms or keywords.

### 2.2 Eligibility Criteria

All RCTs comparing GLP‐1 agonists to placebo or standard therapy for patients with PD were included in the meta‐analysis. Exclusion criteria included all study designs other than RCTs for example, observational studies and animal studies. No language or date restrictions were applied.

### 2.3 Study Selection and Data Extraction

We utilized the software Rayyan to identify and remove duplicate articles from our online literature search. Screening of titles and abstracts was independently carried out by two authors (H.R. and M.A.F.) in order to exclude irrelevant data. Thereafter, the full text screening was done to exclude all articles not matching our inclusion criteria. Any discrepancies were resolved by the senior author (M.E.U.R).

Relevant data were extracted into a pre‐piloted Excel spreadsheet which included: (1) author name and publication year; (2) country; (3) sample size; (4) mean age, in years with standard deviation; (5) percentage proportion of males; (6) Movement Disorder Society Unified Parkinson's Disease Rating Scale (MDS‐UPDRS) Part I (MDS‐UPDRS 1); (7) MDS‐UPDRS 2; (8) MDS‐UPDRS 3; (9) MDS‐UPDRS 4; (10) Parkinson's Disease Questionnaire‐39 (PDQ‐39); (11) Non‐Motor Symptoms Scale for Parkinson's Disease (NMSS); (12) the specific drug, dose, frequency, and duration of GLP‐1 agonists; and (13) control.

### 2.4 Outcomes

The primary outcomes were mean change from baseline in MDS‐UPDRS 3, both on and off medication. The secondary outcomes of our study included mean change from baseline in MDS‐UPDRS 1, MDS‐UPDRS 2, MDS‐UPDRS 4, NMSS, PDQ‐39, as well as nausea, vomiting, constipation, weight loss, diarrhea, and abdominal pain.

### 2.5 Risk of Bias Assessment

Included studies were evaluated for risk of bias using the revised Cochrane Risk of Bias Tool (RoB 2.0). This tool assesses bias across five domains which include: (1) bias arising from the randomization process; (2) bias due to deviation from intended intervention; (3) bias due to missing outcome data; (4) bias in measurement of the outcome; and (5) bias in selection of the reported result.

The risk of bias assessment for each study was conducted by two independent investigators (B.N. and A.N.) with assessments of high risk, low risk or some concerns being reported. Any disagreements were settled by the senior investigator (M.E.U.R).

### 2.6 Data Synthesis

In order to conduct the meta‐analysis, we used the Review Manager (RevMan Version 5.4.1) software. The inverse variance method was used for continuous outcomes, and the Mantel–Haenzsel method for dichotomous outcomes. Mean differences (MD) and risk ratios (RR) were extracted, respectively, along with their corresponding 95% confidence intervals (CI). A random effects model was used to carry out the meta‐analysis. Pooled studies were presented in the form of forest plots; the Higgins *I* ^2^ statistics were calculated for the purpose of assessing heterogeneity between studies.

### 2.7 Certainty of Evidence Assessment

The Grades of Recommendation, Assessment, Development, and Evaluation (GRADE) framework was utilized to assess certainty of evidence. Two authors independently assessed the certainty of evidence (B.N. and A.N.); disagreements were settled by a third author (M.E.U.R.).

## 3 Results

### 3.1 Search Results

**FIGURE 1:**
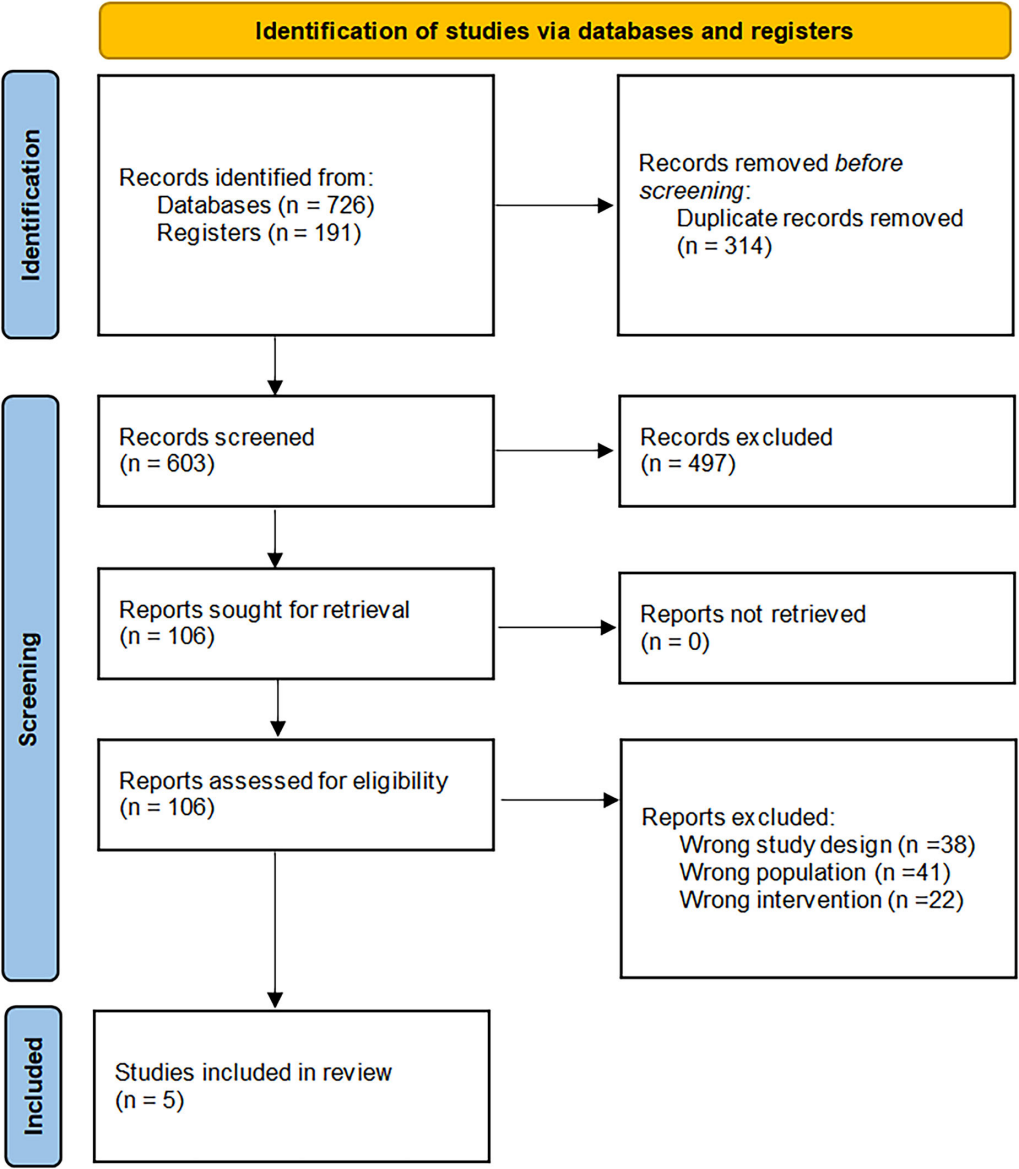
PRISMA flowchart.

Our initial literature search retrieved a total of 917 articles. Of these, 497 articles were excluded during title and abstract screening, followed by exclusion of 101 more during full‐text screening. Consequently, five studies were ultimately included in the meta‐analysis. The detailed screening process is depicted in Figure 1.

### 3.2 Study Characteristics

This meta‐analysis comprised of five RCTs that met the eligibility criteria (Mcgarry et al. 2024; Athauda et al. 2017; Meissner et al. 2024; Aviles‐Olmos et al. 2013; Vijiaratnam et al. 2025). A total of 708 patients were included, out of which 396 patients received GLP‐1RAs and 312 patients received placebo. Three studies used exenatide, one administered NYL01, and one used lixisenatide as GLP‐1 agonists. The publication years ranged from 2013 to 2025, with three studies taking place in the United Kingdom and one each in France and the United States. Study characteristics are reported in Table 1.

**TABLE 1:** Characteristics of included studies.

Study ID	Country	Sample Size	Mean age, in years (SD)	Male (%)	MDS‐UPDRS 1	MDS‐UPDRS 2	MDS‐UPDRS 3	MDS‐UPDRS 4	PDQ39	NMSS	Intervention (drug, dose, frequency, duration)	Control	Outcome assessment timeperiod
Vijiaratnam 2025	United Kingdom	194 (97 vs. 97)	60.7 (9.1)	71	7.9 (4.9) vs. 7.5 (4.8)	7.4 (4.9) vs. 7.5 (4.9)	20.0 (10.2) vs. 20.8 (10.4)	3.9 (3.3) vs. 3.8 (3.2)	11.9 (9.3) vs. 10.6 (8.0)	30.5 (24.9) vs. 28.2 (24.0)	Exenatide: 2 mg once a week for 96 weeks	Placebo	0– 96 weeks
Athauda 2017	United Kingdom	60 (31 vs. 29)	61.6 (8.2) vs. 57.8 (8.0)	73	9.8 (4.8) vs. 9.2 (3.8)	12.5 (6.7) vs. 10.7 (5.3)	19.4 (8.4) vs. 14.4 (8.2)	4.7 (3.1) vs. 5.3 (3.0)	19.9 (13.7) vs. 21.1 (13.0)	24.6 (19.8) vs. 28.3 (24.7)	Exenatide: 2 mg once a week for 60 weeks	Placebo	0–60 weeks
Olmos 2013	United Kingdom	44 (20 vs. 24)	61.4 (6) vs. 59.4 (8.4)	79	10.4 (4.1) vs. 11.6 (4.7)	10.2 (5.2) vs. 12.9 (6.2)	23.5 (6.3) vs. 25.3 (10.7)	6.3 (2.4) vs. 6.3 (3.4)	19.2 (13.5) vs. 24.5 (12.8)		Exanetide: 2 mg once a week for 56 weeks	Placebo	0–12 months
McGarry 2024	United States	254 (170 vs 84)	61.8 (8.1) vs. 62.1 (9.0) vs. 60.6 (10.0)	62 vs. 71 vs. 64	4.7 (4.2) vs. 4.2 (3.1) vs. 4.0 (3.7)	4.9 (3.6) vs. 4.8 (3.6) vs. 5.0 (4.1)	22.3 (9.1) vs. 22.7 (8.1) vs. 22.0 (8.2)		1.7 (0.7) vs. 2.5 (0.7) vs. 1.9 (0.7)	4.1 (1.4) vs. 1.9 (1.5) vs. 1.3 (1.5)	NLY01 2.5 or 5 mg once a week for 36 weeks	Placebo	0–36 weeks
Meissner 2024	France	156 (78 vs. 78)	59.5 (8.1) vs. 59.9 (8.4)	59	6.1 (4.0) vs. 6.4 (4.2)	5.0 (3.5) vs. 5.4 (4.3)	14.8 (7.3) vs. 15.5 (7.8)	0.3 (1.3) vs. 0.2 (0.8)	17.4 (10.9) vs. 16.8 (13)		Lixisenatide: 20 mcg for 12 months (10 mcg for initial 14 days)	Placebo	0–12 months

Abbreviations: MDS‐UPDRS 1 = Movement Disorder Society Unified Parkinson's Disease Rating Scale Part 1, MDS‐UPDRS 1 = Movement Disorder Society Unified Parkinson's Disease Rating Scale Part 2, MDS‐UPDRS 3 = Movement Disorder Society Unified Parkinson's Disease Rating Scale Part 3, MDS‐UPDRS 4 = Movement Disorder Society Unified Parkinson's Disease Rating Scale Part 4, NMSS = Non‐Motor Symptoms Scale for Parkinson's Disease.PDQ‐39 = Parkinson's Disease Questionnaire‐39, SD = Standard deviation.

### 3.3 Risk of Bias Assessment in Included Studies

The quality assessment has been shown in Figure S1. Overall, four studies reported low risk of bias, with only one study from 2013 reporting high risk of bias due to high risk in the selection of reported results paired with some concerns related to deviations from the intended intervention.

### 3.4 Meta‐Analysis of Primary Outcomes

#### 3.4.1 MDS‐UPDRS 3

**FIGURE 2:**
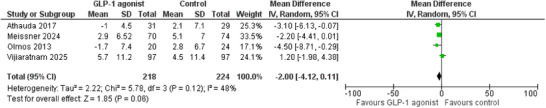
Forest Plot of MDS‐UPDRS 3 off medication.

Four studies were involved in the analysis of MDS‐UPDRS 3 off medication. In total 442 patients (218 GLP‐1RAs vs. 224 placebo) were included. The analysis revealed no statistical significant difference between the two groups (MD: −2.00, 95% CI: −4.12 to 0.11, *p* = 0.06, *I* ^2^ = 48%) (Figure 2). The overall certainty of evidence was evaluated to be moderate (Table S1).

**FIGURE 3:**
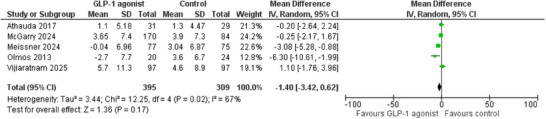
Forest Plot of MDS‐UPDRS 3 on medication.

Five studies were involved in the analysis of MDS‐UPDRS 3 on medication. In total, 704 patients (395 GLP‐1RAs vs. 309 placebo) were included. Our analysis revealed no statistically significant difference between the two groups (MD: −1.40, 95% CI: −3.42 to 0.62, *p* = 0.17, *I* ^2^ = 67%) (Figure 3). The overall certainty of evidence was evaluated to be low (Table S1).

### 3.5 Meta‐Analysis of Secondary Outcomes

#### 3.5.1 MDS‐UPDRS 1

This outcome was reported by all five studies. The total number of patients was 704 patients (395 GLP‐1RAs vs. 309 placebo). The analysis revealed no statistically significant difference in MDS‐UPDRS 1 between both groups (MD: −0.17, 95% CI: −0.99 to 0.65, *p* = 0.69, *I* ^2^ = 41%) (Figure S2). The overall certainty of evidence was evaluated to be moderate (Table S1).

#### 3.5.2 MDS‐UPDRS 2

All five studies reported this outcome. The total comprised of 704 patients (395 GLP‐1RAs vs. 309 placebo). According to our analysis, there was no observable statistically significant difference between the two groups (MD: −0.57, 95% CI: −1.87 to 0.73, *p* = 0.39, *I* ^2^ = 75%) (Figure S3). The overall certainty of evidence was evaluated to be low (Table S1).

#### 3.5.3 MDS‐UPDRS 4

Four of the included studies reported on this outcome. A total of 450 patients (225 GLP‐1RAs vs. 225 placebo) were involved. Our analysis revealed no statistically significant difference between both groups (MD: −0.22, 95% CI: −0.74 to 0.31, *p* = 0.42, *I* ^2^ = 27%) (Figure S4). The overall certainty of evidence was evaluated to be moderate (Table S1).

### 3.6 NMSS

Only three studies reported and were involved in the analysis of NMSS. In total, 508 patients (298 GLP‐1RAs vs. 210 placebo) were included. No statistically significant difference was revealed between both groups according to our meta‐analysis (MD: 0.18, 95% CI: −2.54 to 2.90, *p* = 0.90, *I* ^2^ = 0%) (Figure S5). The overall certainty of evidence was evaluated to be moderate (Table S1).

### 3.7 PDQ‐39

Four reporting studies were included in our meta‐analysis. A total of 552 patients (318 GLP‐1RAs vs. 234 placebo) were involved in total. Our meta‐analysis showed no statistically significant difference between the two groups (MD: −0.34, 95% CI: −1.63 to 0.96, *p* = 0.61, *I* ^2^ = 0%) (Figure S6). The overall certainty of evidence was evaluated to be moderate (Table S1).

### 3.8 Nausea

Nausea was an adverse outcome reported in all five studies. A total of 708 patients (396 GLP‐1RAs vs. 312 placebo) were involved in total for this analysis, which reported statistically significant evidence that taking GLP‐1RAs was associated with greater nausea compared to control groups (RR: 2.09, 95% CI: 1.50–2.91, *p* < 0.0001, *I* ^2^ = 53%) (Figure S7). The overall certainty of evidence was evaluated to be moderate (Table S1).

### 3.9 Vomiting

Four studies were involved in the meta‐analysis of this outcome. This analysis comprised of 664 patients (376 GLP‐1RAs vs. 288 placebo). Our analysis revealed statistically significant difference between the two groups, with GLP‐1RAs having greater association with vomiting (RR: 4.53, 95% CI: 1.95–10.50, *p* = 0.0004, *I* ^2^ = 0%) (Figure S8). The overall certainty of evidence was evaluated to be high (Table S1).

### 3.10 Constipation

Our meta‐analysis included four out of the five studies for this outcome. A total of 552 patients (318 GLP‐1RAs vs. 234 placebo) were involved in total. The results revealed statistically significant difference with GLP‐1RAs having a stronger association with constipation than control groups (RR: 1.60, 95% CI: 1.13–2.27, *p* = 0.008, *I* ^2^ = 26%) (Figure S9). The overall certainty of evidence was evaluated to be high (Table S1).

### 3.11 Weight Loss

All five studies were involved in the meta‐analysis of weight loss. A total of 708 patients (396 GLP‐1RAs vs. 312 placebo) were involved. According to our analysis, GLP‐1RAs were associated with statistically significant weight loss compared to control groups (RR: 1.83, 95% CI: 1.17–2.87, *p* = 0.008, *I* ^2^ = 56%) (Figure S10). The overall certainty of evidence was evaluated to be moderate (Table S1).

### 3.12 Diarrhea

All five studies reported on diarrhea. The total comprised of 708 patients (396 GLP‐1RAs vs. 312 placebo). According to our analysis, there was no observable statistically significant difference between the two groups (RR: 1.45, 95% CI: 0.98–2.15, *p* = 0.06, *I* ^2^ = 0%) (Figure S11). The overall certainty of evidence was evaluated to be moderate (Table S1).

### 3.13 Abdominal Pain

Four reporting studies were included in our meta‐analysis of this outcome. A total of 454 patients (226 GLP‐1RAs vs. 228 placebo) were involved in total. Our meta‐analysis showed no statistically significant difference between the two groups for abdominal pain (RR: 1.53, 95% CI: 0.84–2.78, *p* = 0.16, *I* ^2^ = 0%) (Figure S12). The overall certainty of evidence was evaluated to be moderate (Table S1).

## 4 Discussion

This systematic review and meta‐analysis represent the most comprehensive analyses so far that have examined the effects of GLP‐1RAs on patients with PD, by consolidating data from five RCTs including 709 participants. Our study offers valuable insights into the motor, non‐motor and safety profile of GLP‐1RAs in individuals with PD. Although prior meta‐analyses have been undertaken on this topic, they have included observational data, which is associated with selection bias (Albuquerque et al. 2025; Dahiya et al. 2022; Agid 1991). Our inclusion of only data from RCTs made it possible to assess more pertinently, the therapeutic effects and possible risks of GLP‐1RAs in PD; such as the changes in motor and non‐motor symptoms, quality of life, and adverse events.

Assessment of the motor improvement, which was assessed through MDS‐UPDRS Part 3, showed little and statistically non‐significant outcomes of the patients with GLP‐1 therapy both on and off medication. This nuanced outcome diverges from the previous literature. A study by Athauda et al. (2017) where the authors observed that weekly exenatide intervention resulted in nearly a 4‐point increase in motor scores during the 48‐week intervention

In addition, preliminary results of a study by Aviles‐Olmos et al. (2013) pointed toward possible disease‐modifying effects of GLP‐1 agents. But the same positive findings have not been seen in other, newer‐generation trials using different agents than the US phased studies, including lixisenatide and NLY01, perhaps because of the increase in follow‐up or extend the diversity of the study population, or pharmacodynamics variations between agents. However, our findings, encompassing larger and more recent trials, paint a more conservative picture, possibly due to longer study durations, varying drug molecules, and increased sample heterogeneity. It is worth noting that two recent studies using newer agents (NLY01 and lixisenatide) did not replicate the significant motor gains seen with exenatide (Mcgarry et al. 2024; Meissner et al. 2024; Vijiaratnam et al. 2025).

To fully capture a holistic view of multi‐dimensional response by GLP‐1RAs with PD, we entered into the additional MDS‐UPDRS scales such as Part 1 (non‐motor), Part 2 (motor experiences of daily life), and Part 4, (complications of treatment like dyskinesia). All these analyses also did not find any significant effects of treatment, which is consistent with the findings of Mcgarry et al. (2024); Meissner et al. (2024), and others who had minimal difference found between the active and placebo groups in functional measures and quality‐of‐life dimensions (Vijiaratnam et al. 2025; Mulvaney et al. 2020). Past reviews were constrained by deficient data in many of these subscales and our survey provides a comprehensive and current review.

Other assessment scales including PDQ‐39 and NMSS also revealed that there was a lack of statistically significant enhancement of quality of life and non‐motor burden when there is use of GLP‐1RAs in the treatment of patients. The results are remarkable in the context of the strong‐measured influence of non‐motor symptoms such as disturbances of mood, sleeping disorders, gastrointestinal disorders on disability and quality of life during PD (Cifuentes et al. 2021; Han et al. 2022; Tseng et al. 2025; Monti et al. 2022; Zhao et al. 2021). Despite a few open‐label or early‐phase trials reporting benefits in the fatigue and mood symptoms, the trials are susceptible to placebo outcomes and blinding shortcomings. Also, some instruments like the PDQ‐39 and NMSS might have a low sensitivity to smaller or initial changes in a particular non‐motor area (Meissner et al. 2024; Hogg et al. 2022; Schejter‐Margalit et al. 2022).

Safety results were also evaluated, and our meta‐analysis showed excess gastrointestinal events especially nausea and vomiting, as has been previously reported with diabetes and obesity trials (Cifuentes et al. 2021; Wang et al. 2023; Safar‐Boueri et al. 2021). There was also a higher incidence of diarrhea in the GLP‐1 arm but this was not statistically significant. Although these gastrointestinal problems have been mostly temporary, they may compromise compliance with treatment‐particularly when they affect older populations of patients with PD, who already have an inclination towards developing autonomic dysfunction (Cifuentes et al. 2021). The abdominal pain and flatulence did not show a significant deviation between groups as seen in previous reviews. However, paradoxically, we arrived to the conclusion that there was indeed a strong relationship between the use of GLP‐1 and constipation despite the pro‐motility activity attributed to this class in certain metabolic diseases. However, in PD delayed gastrointestinal transit is already common and GLP‐1 agonists can even increase this process (Han et al. 2022; Monti et al. 2022).

PD patients also experienced weight loss that can be considered a positive effect of GLP‐1 agonists in metabolic syndromes. Although it may be beneficial in patients with overweight, inadvertent weight loss in PD has been observed to predispose sarcopenia, frailty, and abnormally high mortality risk (Cifuentes et al. 2021). Clinicians are thus advised to follow closely the nutritional status of the patients notably those with low body mass index or those with baseline cachexia. Mechanistically the actions of the GLP‐1RAs entail boosting the secretion of insulin in a glucose‐dependent way, slowing down stomach emptying, and adjusting appetites, all of which lead to weight loss as well as indicators of gastrointestinal symptoms (Dahiya et al. 2022; Tseng et al. 2025).

In addition to the peripheral effects, the anti‐neurodegenerative to neuroprotective action of the GLP‐1RAs lies in the central activity. Some of them are able to pass through the blood–brain barrier and reach receptors in areas as substantia nigra, cortex, or hippocampus. Activation of GLP‐1 receptor has been linked to the mitigation of microglial activation, neuroinflammation and mitochondrial improvement and decreased 1‐synuclein aggregation and improved dopaminergic functioning in preclinical models (Schejter‐Margalit et al. 2022). There is also increasing evidence on the relationship between insulin resistance and pathophysiology of PD that contributes to the rationale to use GLP‐1 analogs as disease‐modifying treatment (Sabari et al. 2023; Wikipedia 2025; Kalinderi et al. 2024; Collins and Costello 2025).

Nonetheless, the clinical interpretation of such biological effects is not yet conclusive. Although the GLP‐1RAs remain a promising therapy, particularly where brain‐penetrant neurotherapeutics are involved, the relatively small symptomatic improvements so far reported are not enough to justify its wide adoption in PD, in the absence of clinical trials. Further research needs to be aimed at longer study periods, earlier start time and the next generation GLP‐1 agents that penetrate more in the central nervous system.

The studies included in our analysis employed pharmacologically distinct GLP‐1RAs at varying doses, which may contribute to the heterogeneity observed in our pooled effect sizes. NLY01 is a longer‐lasting pegylated analogue of exenatide, designed primarily for immunomodulation via microglial inhibition rather than direct CNS penetration, while lixisenatide exhibits higher affinity for the GLP‐1 receptor and a more favorable tolerability profile compared to exenatide (Mcgarry et al. 2024; Meissner et al. 2024). These agents therefore differ meaningfully in their pharmacokinetics, CNS penetration, and putative neuroprotective mechanisms. Although class‐level pooling was performed given the shared GLP‐1 receptor agonism and the limited number of trials per individual agent, which precluded meaningful agent‐specific analyses, this represents a major limitation of the present review, and the pooled estimates should be interpreted with caution. Future trials should be adequately powered to enable agent‐specific conclusions and to determine whether particular agents confer differential neuroprotective benefits in PD.

Our work has several limitations. First, despite inclusion of only RCTs to guarantee methodological rigor; study design, trial length, drug, and the subjects of the study might have contributed to heterogeneity within the results. Second, trials seemed to be of moderate duration, which may not be enough to detect long‐term neuroprotective or disease‐modifying effects. Third, non‐motor and quality‐of‐life domains might not have received due representation, possibly due to deficient data or sensitive assessment measures. Fourth, publication bias could not be assessed due to limited number of studies. Fifth, our results might not be applicable to PD patients with comorbidities or unusual disease courses. Sixth, GRADE assessment showed the certainty of evidence was low for two key outcomes, MDS‐UPDRS 3 on medication and MDS‐UPDRS 2, driven by inconsistency and imprecision. Seventh, the included trials did not explicitly report a standardized definition or threshold for weight loss, precluding precise characterization of this outcome across studies and limiting the interpretability of the corresponding pooled estimate.

In conclusion, this systematic review and meta‐analysis evaluated the efficacy and safety of GLP‐1RAs in patients with PD. Available RCT evidence does not demonstrate a statistically significant overall benefit on motor or non‐motor outcomes, while gastrointestinal adverse events were notably increased. Given the uncertainty reflected in the CIs and the heterogeneity across trials, definitive conclusions regarding the suitability of GLP‐1RAs in PD cannot yet be drawn. Longer‐term and larger trials, particularly in earlier stages of disease, may be needed to clarify their disease‐modifying potential and to identify subpopulations that may derive meaningful benefit.

## Author Contributions

**Bakhtmeena Nizam**: conceptualization, investigation, methodology, formal analysis. **Noora Inam**: data curation, writing – review and editing, writing – original draft. **Usman Khan**: conceptualization, writing – review and editing, supervision. **Asma'a Munasar Ali Alsubari**: conceptualization, writing – review and editing, supervision. **Hassan Raza**: conceptualization, methodology, formal analysis, investigation. **Arif Padaniya**: conceptualization, methodology, formal analysis, investigation. **Muhammad Aqib Farid**: conceptualization, methodology, formal analysis, investigation. **Amber Nabi**: conceptualization, methodology, formal analysis, investigation. **Muhammad Qasim Hasan**: data curation, writing – original draft, writing – review and editing. **Muhammad Salih**: data curation, writing – original draft, writing – review and editing. **Apurva Hiteshkumar Patel**: data curation, writing – original draft, writing – review and editing. **Mohammad Ebad Ur Rehman**: data curation, writing – original draft, writing – review and editing, supervision.

## Conflicts of Interest

The authors declare no conflicts of interest.

## Funding

The authors have nothing to report.

## Ethics Statement

This project was exempt from IRB approval on account of being a systematic review.

## Supporting information




**Supplementary Figure 1**: Risk of Bias in RCTs

**Supplementary Figure 2**: Forest Plot of MDS‐UPDRS 1

**Supplementary Figure 3**: Forest Plot of MDS‐UPDRS 2

**Supplementary Figure 4**: Forest Plot of MDS‐UPDRS 4

**Supplementary Figure 5**: Forest Plot of NMSS

**Supplementary Figure 6**: Forest Plot of PDQ39

**Supplementary Figure 7**: Forest Plot of Nausea

**Supplementary Figure 8**: Forest Plot of Vomiting

**Supplementary Figure 9**: Forest Plot of Constipation

**Supplementary Figure 10**: Forest Plot of Weight Loss

**Supplementary Figure 11**: Forest Plot of Diarrhea

**Supplementary Figure 12**: Forest Plot of Abdominal Pain

**Supplementary Table**: brb371344‐supp‐00013‐TableS1.docx

## Data Availability

The data supporting this study's findings are available from the corresponding author upon reasonable request.
