# Supplementary figures and images for: Efficacy and Safety of Glucagon‐Like Peptide 1 Receptor Agonists in Parkinson Disease: A Systematic Review and Meta‐Analysis
Source: Brain Behav. 2026 Mar 25;16(4):e71344. doi: 10.1002/brb3.71344 (PMC13109035; doi:10.1002/brb3.71344)

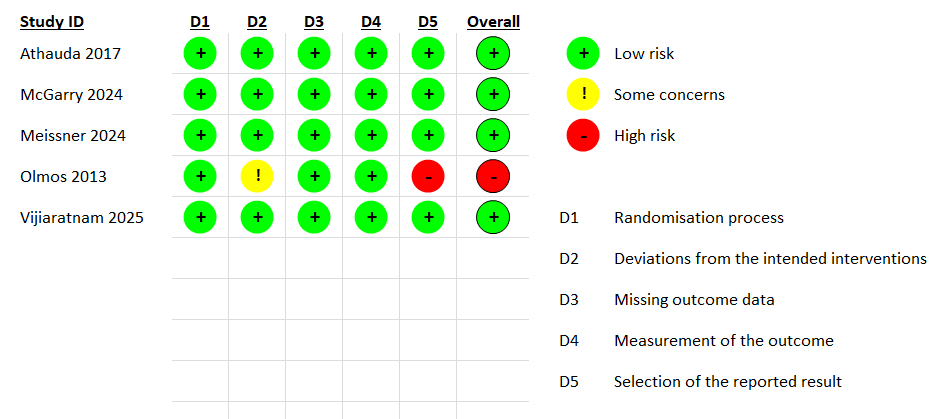

Supplement: Supplementary file 1 — Supplementary Figure 1: Risk of Bias in RCTs [file BRB3-16-e71344-s010.png]

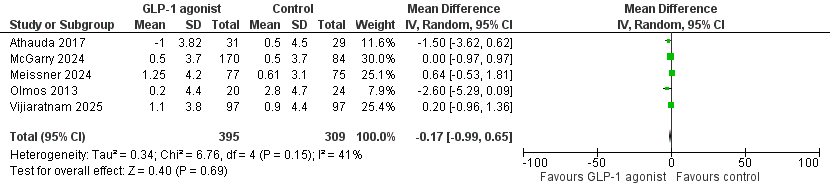

Supplement: Supplementary file 2 — Supplementary Figure 2: Forest Plot of MDS‐UPDRS 1 [file BRB3-16-e71344-s012.png]

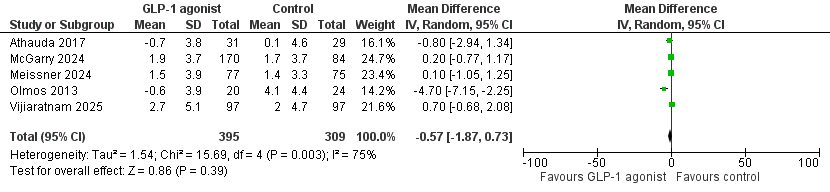

Supplement: Supplementary file 3 — Supplementary Figure 3: Forest Plot of MDS‐UPDRS 2 [file BRB3-16-e71344-s004.png]

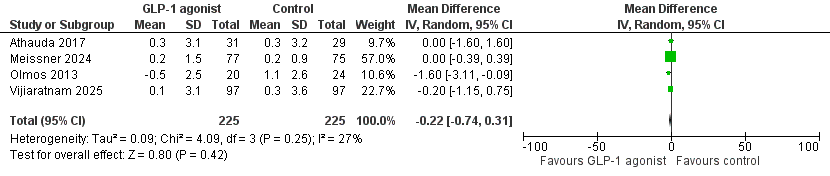

Supplement: Supplementary file 4 — Supplementary Figure 4: Forest Plot of MDS‐UPDRS 4 [file BRB3-16-e71344-s007.png]

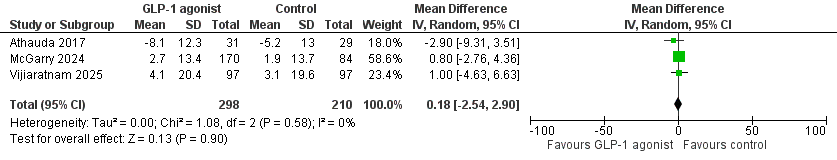

Supplement: Supplementary file 5 — Supplementary Figure 5: Forest Plot of NMSS [file BRB3-16-e71344-s001.png]

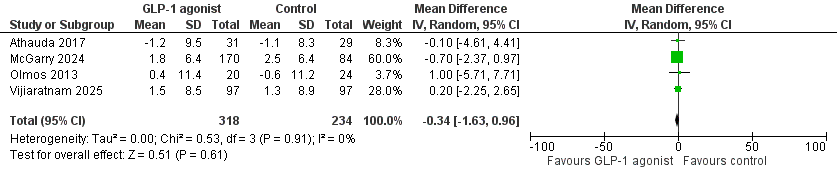

Supplement: Supplementary file 6 — Supplementary Figure 6: Forest Plot of PDQ39 [file BRB3-16-e71344-s011.png]

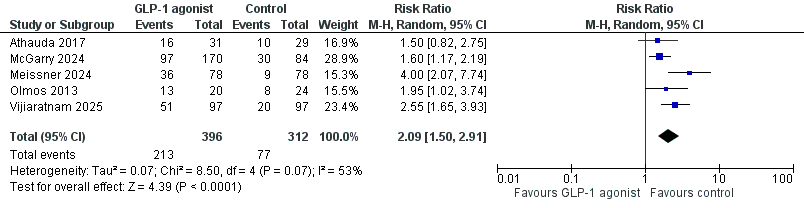

Supplement: Supplementary file 7 — Supplementary Figure 7: Forest Plot of Nausea [file BRB3-16-e71344-s008.png]

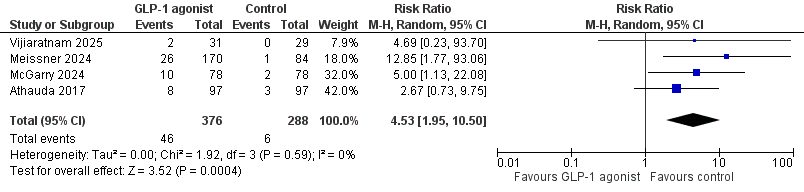

Supplement: Supplementary file 8 — Supplementary Figure 8: Forest Plot of Vomiting [file BRB3-16-e71344-s013.png]

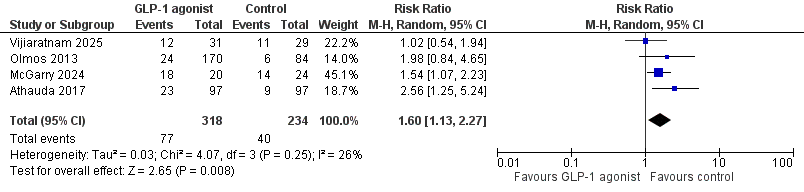

Supplement: Supplementary file 9 — Supplementary Figure 9: Forest Plot of Constipation [file BRB3-16-e71344-s009.png]

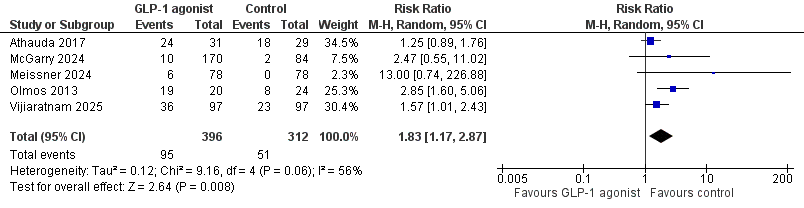

Supplement: Supplementary file 10 — Supplementary Figure 10: Forest Plot of Weight Loss [file BRB3-16-e71344-s006.png]

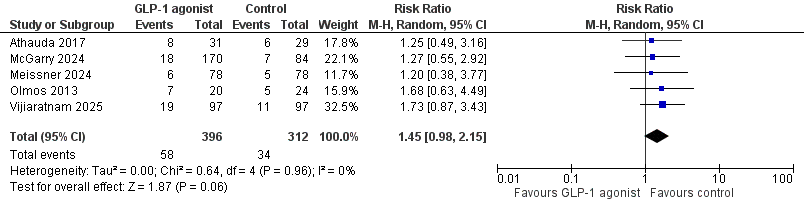

Supplement: Supplementary file 11 — Supplementary Figure 11: Forest Plot of Diarrhea [file BRB3-16-e71344-s005.png]

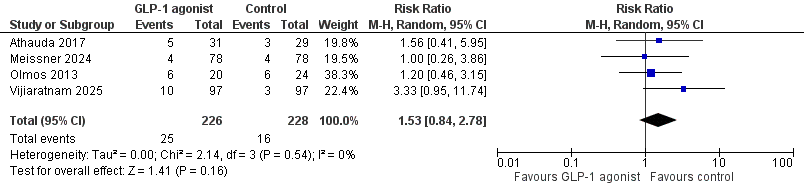

Supplement: Supplementary file 12 — Supplementary Figure 12: Forest Plot of Abdominal Pain [file BRB3-16-e71344-s003.png]
