# Supplementary material for: Efficacy and Safety of Glucagon‐Like Peptide 1 Receptor Agonists in Parkinson Disease: A Systematic Review and Meta‐Analysis
Source: Brain Behav. 2026 Mar 25;16(4):e71344. doi: 10.1002/brb3.71344 (PMC13109035; doi:10.1002/brb3.71344)
Supplement: Supplementary file 13 — Supplementary Table: brb371344‐supp‐00013‐TableS1.docx [file BRB3-16-e71344-s002.docx]

| **Outcome** | **No. of participants (studies)** | **Effect estimates (95% CI)** | **Risk of bias** | **Inconsistency** | **Indirectness** | **Imprecision** | **Publication bias** | **Quality** |
| --- | --- | --- | --- | --- | --- | --- | --- | --- |
| MDS-UPDRS 3 OFF Medication | 442 (4) | MD: -2.00 (-4.12-0.11) | Not serious | Not serious | Not serious | Serious | NA | Moderate |
| MDS-UPDRS 3 ON Medication | 704 (5) | MD: -1.40 (-3.42-0.62) | Not serious | Serious | Not serious | Serious | NA | Low |
| MDS-UPDRS 1 | 704 (5) | MD: -0.17 (-0.99-0.65) | Not serious | Not serious | Not serious | Serious | NA | Moderate |
| MDS-UPDRS 2 | 704 (5) | MD: -0.57 (-1.87-0.73) | Not serious | Serious | Not serious | Serious | NA | Low |
| MDS-UPDRS 4 | 450 (4) | MD: -0.22 (-0.74-0.31) | Not serious | Not serious | Not serious | Serious | NA | Moderate |
| NMSS | 508 (3) | MD: 0.18 (-2.54-2.90) | Not serious | Not serious | Not serious | Serious | NA | Moderate |
| PDQ-39 | 552 (4) | MD: -0.34 (-1.63-0.96) | Not serious | Not serious | Not serious | Serious | NA | Moderate |
| Nausea | 708 (5) | RR: 2.09 (1.50-2.91) | Not serious | Serious | Not serious | Not serious | NA | Moderate |
| Vomiting | 664 (4) | RR: 4.53 (1.95-10.50) | Not serious | Not serious | Not serious | Not serious | NA | High |
| Constipation | 552 (4) | RR: 1.60 (1.13-2.27) | Not serious | Not serious | Not serious | Not serious | NA | High |
| Weight Loss | 708 (5) | RR: 1.83 (1.17-2.87) | Not serious | Serious | Not serious | Not serious | NA | Moderate |
| Diarrhea | 708 (5) | RR: 1.45 (0.98-2.15) | Not serious | Not serious | Not serious | Serious | NA | Moderate |
| Abdominal Pain | 454 (4) | RR: 1.53 (0.84-2.78) | Not serious | Not serious | Not serious | Serious | NA | Moderate |

Supplementary Table 1: Grading of recommendations assessment, development, and evaluation (GRADE) summary of findings
